# Superior survival benefits of triple combination immunotherapy compared to standard chemotherapy as second-line treatment for advanced biliary tract cancer: a retrospective analysis

**DOI:** 10.3389/fonc.2024.1482909

**Published:** 2024-11-22

**Authors:** Peipei Shang, Heming Xu, Tianmei Zeng, Cheng Lou, Wei Wei, Guang Yang, Zhuo Cheng, Xiaowen Cui, Weipeng Hong, Weidong Shen, Zhicong Lian, Zhengang Yuan

**Affiliations:** ^1^ Eastern Hepatobiliary Surgical Hospital, Naval Military Medical University, Shanghai, China; ^2^ Medical Department, Oyeah Biotech, Shanghai, China; ^3^ Department of Marketing, Singlera Genomic Ltd., Shanghai, China

**Keywords:** BTC, second-line therapy, immunotherapy, combination (combined) therapy, retrospective studies

## Abstract

**Background:**

Advanced biliary tract cancer (BTC) is associated with a poor prognosis and limited options for second-line treatment. The TOPAZ-1 and KEYNOTE-966 trials have demonstrated the benefits of combining immune checkpoint inhibitors (ICIs) with chemotherapy in treating BTC. However, the efficacy of FOLFOX as a second-line therapy is limited, highlighting the need for more effective treatment approaches.

**Methods:**

This retrospective study compared a triple regimen—comprising ICIs, tyrosine kinase inhibitors, and chemotherapy—to standard chemotherapy in patients with metastatic BTC who had progressed on first-line gemcitabine-based therapy. The primary endpoint was progression-free survival (PFS), with secondary endpoints including overall survival (OS), overall response rate (ORR), disease control rate (DCR), and safety.

**Results:**

Of the 121 patients, 86 received the triple regimen and 35 received standard chemotherapy. The triple regimen showed a significantly higher ORR (37.2% vs. 2.8%, p < 0.0001) and DCR (89.5% vs. 71.4%). The median PFS was 6 months for the triple regimen compared to 2.0 months for standard chemotherapy (HR 0.29, p < 0.0001). The median OS was 16.0 months for the triple regimen versus 6.0 months for standard chemotherapy (HR 0.35, p < 0.0001). Treatment-related adverse events were comparable between the groups.

**Conclusion:**

The triple combination of immunotherapy offers superior survival benefits compared to standard chemotherapy as a second-line treatment for advanced BTC, warranting further investigation for potential clinical adoption.

## 1 Introduction

Biliary tract cancer (BTC), encompassing both gallbladder cancer (GBC) and cholangiocarcinoma (CCA), represents a highly aggressive and therapeutically challenging group of malignancies. Characterized by significant heterogeneity and a complex tumor microenvironment, these cancers complicate early diagnosis and are associated with a propensity for chemoresistance (1–3). The incidence and mortality rates of BTC are rising, and affected patients face a poor prognosis. CCA is further classified into perihilar, distal, and intrahepatic subtypes based on anatomical location, exhibiting variations in etiology, epidemiology, pathogenesis, diagnosis, and treatment. For example, among CCA patients treated with the standard chemotherapy regimen of gemcitabine and cisplatin, the median survival time following early detection is approximately 24 months, which decreases to 11.7 months in cases of metastatic disease (2, 4). GBC, the most aggressive form of BTC, shows variability in median survival times based on geographical region, with the 5-year survival rate ranging from 80% for stage 0 to as low as 2% for stage IVB, according to data from the National Cancer Database.

Advanced BTC treatment primarily relies on systemic therapies. The gemcitabine and cisplatin combination regimen, known as the GC regimen, has been established as the first-line standard of care for advanced BTC based on results from the Phase III ABC-02 study (5). This regimen demonstrated a significant improvement in overall survival (OS) compared to gemcitabine alone, without increased toxicity. With the advent of immunotherapy, immune checkpoint inhibitors have increasingly been explored for liver cancer treatment. Recent trials, including TOPAZ-1 and KEYNOTE-966, have shown that combining standard first-line chemotherapy with immune checkpoint inhibitors (ICIs) targeting programmed cell death protein-1 (PD-1) or programmed death-ligand 1 (PD-L1) can enhance survival in patients with untreated metastatic or unresectable CCA (5–7). However, options for second-line treatment of advanced BTC remain limited, and the improvement in OS with the FOLFOX regimen (a combination of fluorouracil, oxaliplatin, and leucovorin) based on the Phase III ABC-06 study was not significant (8, 9).

Therefore, this study aimed to investigate the efficacy of triple regimen therapy involving ICIs, multi-targeted tyrosine kinase inhibitors (TKIs), and chemotherapy as a second-line treatment for advanced BTC. We seek to provide additional therapeutic options and insights into managing advanced BTC to enhance treatment outcomes and improve patient survival and quality of life.

## 2 Methods

### 2.1 Population and data collection

Retrospective data collection involved reviewing the medical records of patients with postoperative metastatic or unresectable BTC who experienced disease progression on first-line therapy at our institution between October 2021 and October 2023. Data acquisition included telephone follow-ups and a thorough examination of medical records. The inclusion criteria were as follows: (1) confirmed pathological diagnosis of gallbladder cancer or cholangiocarcinoma, (2) ineligibility for surgical resection, (3) inability to tolerate or progression after first-line treatment, (4) presence of measurable lesions as per the RECIST 1.1 evaluation criteria, and (5) availability of complete clinical and follow-up data. Staging was performed according to the eighth edition of the American Joint Committee on Cancer (AJCC) Staging Criteria. The exclusion criteria were as follows: (1) patients with cardiac, pulmonary, or hepatic and renal dysfunction and (2) patients with severe or life-threatening complications.

### 2.2 Endpoint of observation

The primary endpoint was time to progression, and the secondary endpoints included OS, objective response rate (ORR), disease control rate (DCR), and incidence of adverse events. The ORR was evaluated by dividing the number of patients achieving complete response (CR) and partial responses (PR) by the total number of patients. The DCR was calculated by dividing the total number of patients exhibiting CR, PR, and stable disease (SD) by the total number of patients.

### 2.3 Follow-up and efficacy evaluation

During treatment, the patients were evaluated every 2–3 months using abdominal enhanced CT, chest enhanced CT, and other tests. The RECIST 1.1 criteria were used for efficacy evaluation. Patients underwent safety follow-ups after treatment once every three weeks. After discharge, follow-up was conducted via telephone, WeChat, and outpatient visits.

### 2.4 Statistical analysis

GraphPad Prism 9 was used for the data analysis. Quantitative data were expressed as medians. The Kaplan–Meier method was used to plot survival curves, and the log-rank test was used to compare differences between groups. P < 0.05 was considered statistically significant.

## 3 Results

A total of 121 patients were included in this study, with their baseline data and treatment details presented in **Table 1**. Of these, 86 received the triple regimen as a second-line treatment, while 35 received standard chemotherapy. The triple regimen, termed the Tricom Cohort, consisted of tislelizumab or sintilimab (200 mg, administered every three weeks), combined with capecitabine (1500 mg, taken twice daily orally from days 1 to 14, followed by a one-week break), and tyrosine kinase inhibitors (TKIs), specifically lenvatinib (8 mg, taken daily) or anlotinib (10 mg, taken daily from days 1 to 14, followed by a one-week break). The standard chemotherapy group, referred to as the Standard Cohort, included mFOLFOX6.

**Table 1:** Demographic and disease characteristics at baseline.

	Tricom cohort (n=86)	Standard cohort (n=35)
Sex		
Male	39 (45%)	24 (69%)
Female	47 (55%)	11 (31%)
Age (year)	60 (36~82)	57 (39~72)
ECOG PS		
0	16 (19%)	0 (0%)
1	68 (78%)	34 (97%)
2	2 (3%)	1 (3%)
Primary site		
Gall bladder	23 (27%)	6 (17%)
Biliary duct	63 (73%)	29 (83%)
CEA		
>5	35 (40%)	21 (60%)
CA199		
>37	54 (63%)	27 (77%)
Distant metastasis		
Yes	86 (100%)	34 (97%)

There were no significant differences between the two groups in terms of sex, age, ECOG performance status, primary tumor site, levels of carcinoembryonic antigen (CEA) and carbohydrate antigen 199 (CA199), and the presence of distant metastasis (**Table 1**).

According to the RECIST 1.1 criteria, among the 86 patients treated with the triple regimen, 1 patient (1.2%) had CR; 31 (36.0%), PR with significant tumor shrinkage; 45 (52.3%), SD; and, 9 (10.5%), progressive disease (PD). The ORR was 37.2%, and the DCR was 89.5%. Among the 35 patients receiving standard chemotherapy, none achieved CR; 1 (2.8%), PR; 24 (68.6%), SD; and, 10 (28.6%), PD (**Table 2**). The ORR was 2.8%, and the DCR was 71.4%. The ORR between the two groups differed significantly (p < 0.0001) (**Table 2**).

**Table 2:** Tumor response.

	Tricom cohort (n=86)	Standard cohort(n=35)
ORR	32 (37.2%)	1 (2.8%)
CR	1 (1.2%)	0 (0%)
PR	31 (36.0%)	1 (2.8%)
SD	45 (52.3%)	24 (68.6%)
PD	9 (10.5%)	10 (28.6%)
DCR	77 (89.5%)	25 (71.4%)

**Figure 1:**
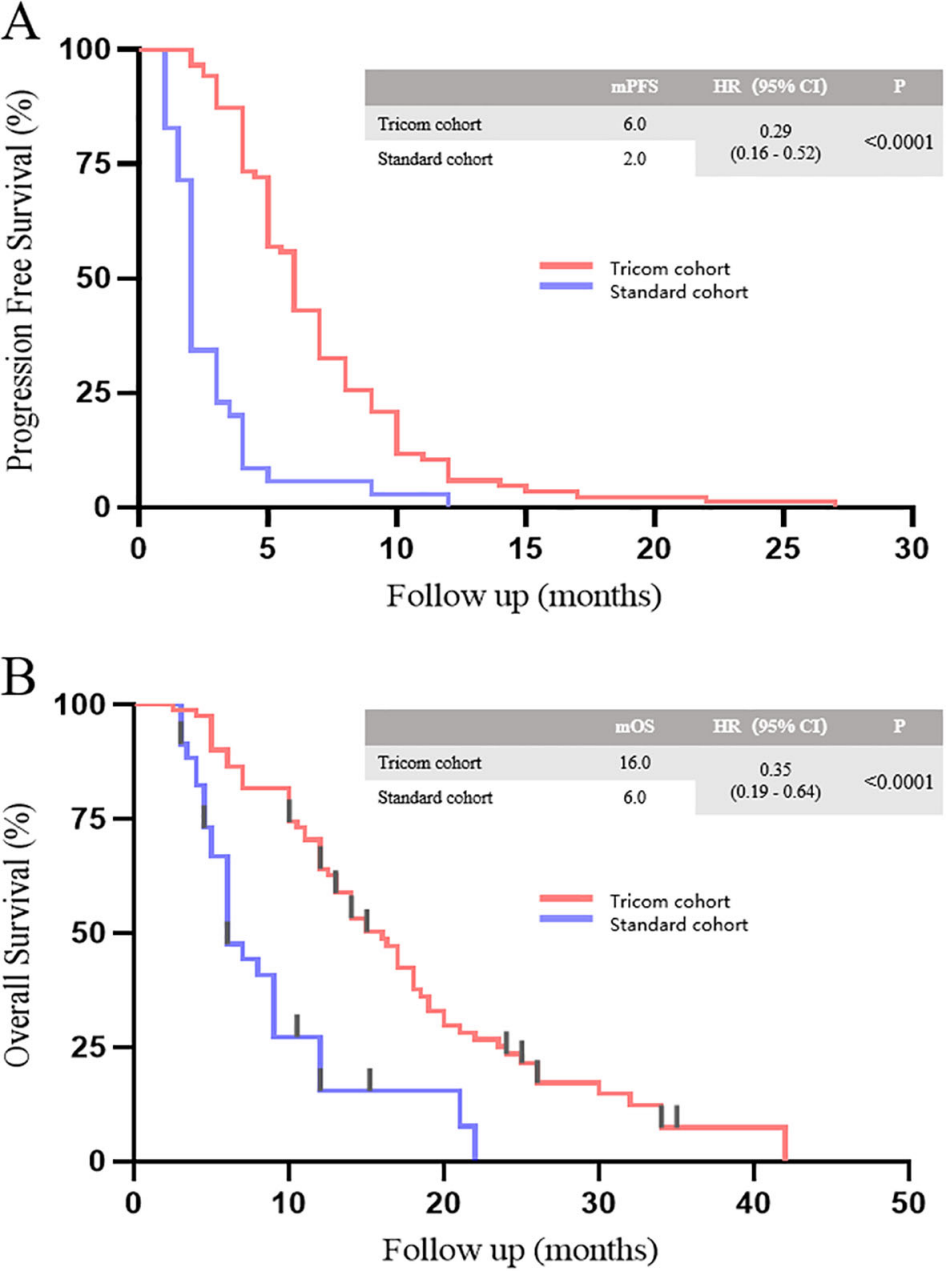
PFS **(A)** and OS **(B)** between tricom and standard cohort.

As of October 31, 2023, all patients in the tricom and standard cohorts had PD. The median progression-free survival (PFS) in the tricom and standard cohort was 6 and 2 months respectively, indicating a significant prolongation of PFS with the triple regimen (95% CI HR=0.29 [0.16–0.52], p<0.0001; **Figure 1A**). In the tricom cohort, 64 patients died and 4 were lost to follow-up, whereas in the standard cohort, 28 patients died. The median OS in the tricom and standard cohort was 16 and 6 months, respectively, showing a significant improvement in OS with the triple regimen (95% CI HR=0.35 [0.19–0.64], p<0.0001. **Figure 1B**).

In terms of safety, 47 patients (54.6%) in the tricom cohort experienced adverse events of varying degrees, with 17 patients (19.8%) experiencing grade 3 or higher adverse events. These included liver dysfunction in 23 patients (19.8%), with 10 cases (11.6%) being grade 3 or higher; hand–foot syndrome in 16 patients (19.0%), with 6 cases (7.0%) being grade 3 or higher; gastrointestinal reactions in 12 patients (13.5%), with 1 case (1.2%) being grade 3 or higher; hematological toxicity in 13 patients (14.6%); and, thyroid function decline, 13 patients (14.6%). Fifteen patients (42.9%) in the standard chemotherapy group experienced adverse events of varying degrees, with 4 patients (11.5%) having grade 3 or higher adverse events (**Table 3**).

**Table 3:** Treatment-related adverse events.

Events, n (%)	Tricom cohort (n=86)		Standard cohort(n=35)	
	Any grade	Grade≥3	Any grade	Grade≥3
All	47 (54.7%)	17 (19.8%)	15 (42.9%)	4 (11.5%)
Hypohepatia	23 (25.8%)	10 (11.6%)	8 (22.9%)	3 (8.6%)
Hand-foot syndrome	16 (19%)	6 (7.0%)	5 (14.3%)	1 (2.9%)
Gastrointestinal reaction	12 (13.5%)	1 (1.2%)	3 (8.6%)	0
Hematologic toxicity	13 (14.6%)	0	3 (8.6%)	0
hypothyroidism	13 (14.6%)	0	3 (8.6%)	0

## 4 Discussion

Surgical resection remains the cornerstone of curative treatment for BTC. However, most patients are diagnosed at an advanced stage, which precludes surgical intervention. Consequently, first-line therapy for advanced BTC has shifted from solely chemotherapy to a combination of ICIs and chemotherapy (3, 10). There is no consensus on the preferred second-line treatment. Although standard chemotherapy is frequently used, its efficacy is limited, as demonstrated by a median OS of only 6.2 months in the FOLFOX treatment arm of the ABC-06 study (1, 2). BTC exhibits significant heterogeneity, with diverse origins and alterations in key genes and signaling pathways driving tumorigenesis. Isocitrate dehydrogenase 1 and fibroblast growth factor receptor are promising targets for advanced intrahepatic cholangiocarcinoma, indicating that targeted therapy is becoming a central component of BTC treatment (11). However, only approximately 30% of patients with BTC harbor targetable mutations. Overexpression of vascular endothelial growth factor, epidermal growth factor receptor, and platelet-derived growth factor receptor is common in BTC and correlates with a poor prognosis, making multi-targeted TKI an effective therapeutic approach (12–16). Current guidelines from the NCCN and CSCO recommend multi-target inhibitors or their combination with ICI as a category 2B recommendation for second-line treatment (1, 2).

This study innovatively explored a triple regimen comprising tyrosine kinase inhibitors (TKIs), ICIs, and chemotherapy, achieving a DCR of 89.5%, an ORR of 37.2%, a median PFS of 6 months, and a median OS of 16 months. Compared to the standard chemotherapy group, the triple regimen demonstrated significant therapeutic benefits. In contrast, the median PFS in the ABC-06 study was 2.9 months, which was similar to the median PFS in the standard chemotherapy group of this study. Several small-sample studies have investigated the efficacy of TKI monotherapy or combination therapy with ICI as a second-line BTC treatment (7, 17–21). Notably, phase 2 trials using lenvatinib or anlotinib demonstrated modest efficacy in terms of ORR and PFS (11). These results underscore the potential of both monotherapy with TKI and combination therapy with ICI as second-line BTC treatments. However, caution is warranted while interpreting efficacy data owing to variations in baseline tumor characteristics and types across studies.

In this study, patients in the triple therapy group experienced immune-related adverse events, including grade 1 or 2 rash, hypothyroidism, and diarrhea, which were in line with previous reports. These reactions were largely manageable and effectively controlled through vigilant management strategies and ongoing monitoring (19, 22–24). We conducted baseline screening before treatment and implemented regular monitoring during treatment, including hematological parameters, thyroid function tests, and skin condition observations, to ensure the timely detection and management of any adverse events. Our management strategies included closely observing symptoms, timely adjusting medication dosages, and providing necessary symptomatic treatments, such as using topical corticosteroids or antihistamines to treat rashes, thyroid hormone replacement for hypothyroidism, and antidiarrheal drugs and dietary adjustments to manage diarrhea. These measures effectively controlled most adverse events, ensuring patient safety and continuity of treatment. Furthermore, although the incidence of adverse events was higher in the triple therapy group, these events did not significantly impact patients’ quality of life, possibly due to our timely interventions and proactive management strategies. Our findings underscore the importance of baseline screening and regular monitoring when implementing triple therapy and demonstrate the potential of triple therapy in the treatment of advanced biliary tract tumors, providing valuable experience and insights for future research and clinical practice.

Despite its contributions, this study has limitations inherent to its retrospective design. Although this study highlights the favorable tumor response to the triple regimen, further validation through rigorous prospective studies is imperative. Moreover, while the triple regimen enhanced the ORR, biomarkers associated with its efficacy warrant further exploration. These limitations offer valuable insights into the design and execution of future clinical trials aimed at elucidating the efficacy of a triple combination regimen as a viable second-line treatment for advanced BTC.

In conclusion, the retrospective analysis underscores the superior efficacy of triple combination immunotherapy over standard chemotherapy in advanced BTC, with notable improvements in progression-free and overall survival. These findings suggest a promising therapeutic advancement, warranting further prospective research to validate these results and potentially redefine second-line treatment strategies for BTC patients.

## Data Availability

The original contributions presented in the study are included in the article/supplementary material. Further inquiries can be directed to the corresponding author.
